# High‐Precision Hemodynamic and Echocardiographic Assessment of Pacing in Obstructive Hypertrophic Cardiomyopathy

**DOI:** 10.1111/pace.70024

**Published:** 2025-08-21

**Authors:** Jagdeep S. Mohal, Matthew J. Shun‐Shin, Kayla Chiew, Alejandra A. Miyazawa, Florentina Simader, Pannathorn Tangkongpanich, Rahul Bahl, Ji‐Jian Chow, James P. Howard, Akriti Naraen, Keenan Saleh, Jack W. Samways, Daniel Keene, S. M. Afzal Sohaib, Mark Tanner, Kevin M. W. Leong, Norman A. Qureshi, David C. Lefroy, Prapa Kanagaratnam, Darrel P. Francis, Amanda Varnava, Zachary I. Whinnett, Ahran D. Arnold

**Affiliations:** ^1^ National Heart and Lung Institute Imperial College London Hammersmith Hospital London UK; ^2^ Barts Heart Centre St Bartholomew's Hospital West Smithfield London UK; ^3^ National University Heart Centre National University Hospital Singapore

**Keywords:** hemodynamics, hypertrophic cardiomyopathy, obstructive hypertrophic cardiomyopathy, right ventricular pacing

## Abstract

**Background:**

Left ventricular outflow obstruction drives symptoms and outcomes in obstructive hypertrophic cardiomyopathy (oHCM). Right ventricular pacing (RVP) can desynchronize the left ventricle to relieve this and allows control of atrioventricular delay (AVD) but may impair ventricular function. We used high‐precision assessment to quantify the hemodynamic and echocardiographic effects of RVP in oHCM.

**Methods:**

Patients with oHCM and implanted dual‐chamber pacing devices underwent continuous recording of ECG, beat‐by‐beat outflow tract continuous wave Doppler, and beat‐by‐beat, noninvasive finger‐cuff blood pressure while pacing was alternated between atrium‐only pacing and AV‐sequential RVP at a range of AVDs at 5 bpm above resting heart rate and 100 bpm. Changes in systolic blood pressure (∆SBP) and left ventricular outflow tract gradient (∆LVOTg) were fitted to parabolas to produce reproducible, narrow confidence interval estimates of effects.

**Results:**

Twenty two patients were recruited (60% male, mean resting LVOTg 53 mmHg). At just above resting heart rate (mean 75 bpm), RVP produced mean peak ∆SBP from AAI to DDD of 2.47 mmHg (95% confidence interval: 0.19–4.76, *p* = 0.04). The mean AVD for peak ∆SBP was 173.2 ms. Mean LVOTg reduction at this AVD was 8.31 mmHg (2.43–14.18, *p* < 0.001). Apart from the hemodynamically optimum AVD, no other AVDs produced statistically significant increases in SBP. At 100 bpm, greater increases in SBP and reductions in LVOTg were seen at hemodynamically optimal AVD.

**Conclusion:**

Multiple alternation assessment allows precise, reproducible, narrow confidence interval quantification of hemodynamic and echocardiographic pacing effects. RVP can reduce LVOTg while preserving or improving cardiac output, but AVD is a key modifier of this relationship.

## Graphical abstract

In obstructive hypertrophic cardiomyopathy (oHCM), right ventricular pacing (RVP) can desynchronize the left ventricle to relieve outflow tract obstruction and allow control of atrioventricular delay (AVD), but may impair ventricular function. In twenty two patients with oHCM, a high‐precision RVP protocol quantified outflow tract gradient (LVOTg) and systolic blood pressure (SBP) effects. Peak ∆SBP from AAI to DDD was 2.47 mmHg (0.19–4.76, *p* = 0.04). LVOTg reduction at optimal AVD was 8.31 mmHg (2.43–14.18, *p* < 0.001). Only the hemodynamically optimal AVD produced a statistically significant SBP increase. RVP can reduce LVOTg while preserving or improving cardiac output; AVD is a key modifier of this relationship.

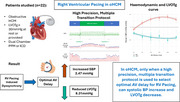

**Abbreviations**
AV: atrioventricular
ECG: electrocardiograph
HCM: hypertrophiccardiomyopathy
LVOTg: left ventricular outflow tract gradient
LVOTO: left ventricular outflow tract obstruction
oHCM: obstructive hypertrophic cardiomyopathy

## 1 Introduction

Left ventricular outflow tract obstruction (LVOTO) drives symptoms and outcomes in obstructive hypertrophic cardiomyopathy (oHCM) [1]. Right ventricular pacing (RVP) can desynchronize left ventricular (LV) activation to delay lateral wall contraction relative to the septum to prevent narrowing of the LV outflow tract during systole [2], thereby reducing the pressure gradient across the LVOT (LVOTg) [3]. RVP‐induced dyssynchrony also impairs myocardial contraction force and dual chamber atrio‐ventricular sequential RVP allows control of AV delay (AVD), the time between atrial and ventricular contraction. These effects must be balanced correctly for RVP to produce a symptomatic benefit to a patient [4]. If RVP reduces LVOTg in a hemodynamically deleterious way, impairing cardiac output [5], it is unlikely that patients will gain benefit from pacing. To assess whether individual patients gain hemodynamic benefit from RVP, a high‐precision assessment of pacing effects is necessary to overcome noise and bias [6, 7].

A number of strategies for selecting programmed AVD have been implemented historically: ultrashort AVD, longest possible AVD, AVD producing the lowest LVOTg, and AVD reducing LVOTg without impairing aortic pressure [8]. One aspect of AVD selection is constant between these historical strategies: low‐precision methodology, prone to bias and noise, to assess their effects and thereby determine which strategy optimizes pacing [9].

In this prospective study, we set out to apply high‐precision methodology to quantify the hemodynamic and echocardiographic effects of RVP and determine optimal AVD.

## 2 Methods

### 2.1 Study Population

Patients with oHCM and implanted dual chamber pacing devices under follow‐up at a single tertiary cardiac center (Hammersmith Hospital, London, UK) were screened for eligibility. Inclusion criteria for the study were as follows: (i) resting LVOTg ≥ 30 mmHg, (ii) < 20% ventricular pacing burden, (iii) sinus rhythm, (iv) non‐LBBB intrinsic QRS morphology, (v) able to give informed consent, and (vi) age ≥ 18. All participants gave written, informed consent and the study was approved by the local ethics committee (21/WS/0169).

### 2.2 Acute Hemodynamic and Echocardiographic Study

The following were recorded continuously throughout the study via a data acquisition system (National Instruments, USA) using custom software:

- Noninvasive beat‐by‐beat finger‐cuff blood pressure via Finometer (Finapres NOVA, Finapres Medical Systems, Netherlands)
- Beat‐by‐beat continuous wave LVOT Doppler via transthoracic echocardiography (Epiq CVx, Phillips, Netherlands)
- Limb‐lead ECG (Dynascope DS‐7100, Fukuda Denshi, Japan)

Pacing‐induced changes in noninvasive beat‐by‐beat blood pressure have previously been validated for acute hemodynamic assessment [10]. The instantaneous change in systolic blood pressure (SBP) excludes vascular resistance changes, which take time to develop, meaning ∆SBP is directly proportional to the change in cardiac output [11]. We used a validated high‐precision method for determining the peak hemodynamic response to RVP, which has been described in detail previously and summarized here [12].

**FIGURE 1:**
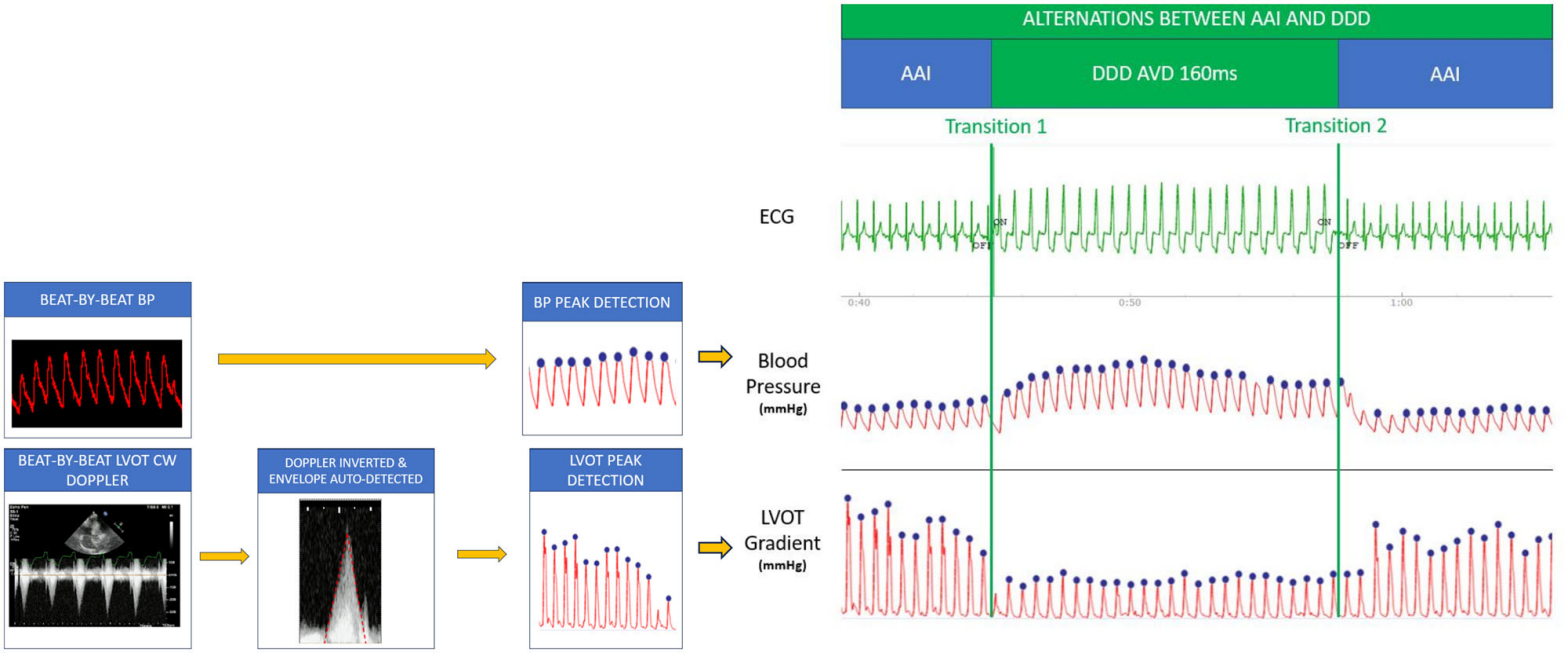
Acquisition of hemodynamic and echocardiographic data. Continuously acquired beat‐by‐beat noninvasive blood pressure waveforms undergo automated peak detection to determine systolic blood pressure data points for each beat's waveform. Continuous wave Doppler through the left ventricular outflow tract is continuously acquired using screen capture. The Doppler envelope is inverted and autotraced to determine left ventricular outflow tract gradient data points for each beat. Pacing is alternated between atrium‐only pacing (AAI—blue) and atrioventricular sequential pacing (DDD—green) multiple times for each tested atrioventricular delay. A range of atrioventricular delays are tested for each heart rate. Two heart rates are tested: just above sinus rate and 100 beats per minute. [Colour figure can be viewed at wileyonlinelibrary.com]

Pacing was alternated between reference (atrium‐only pacing, AAI) and test (atrioventricular sequential pacing, DDD) states multiple times for a given AVD at a full range of AVDs. The tested AVDs ranged from 40 ms until pure intrinsic conduction, in 40‐ms increments. The pacing rate was fixed initially at approximately 5 beats per minute above the sinus heart rate and the whole protocol was repeated at 100 beats per minute. The hemodynamic assessment was performed as follows. Eight beats of blood pressure data were acquired and averaged during AAI pacing (unpaced QRS complex) immediately before transitioning to AV‐sequential RV pacing. Following the transition, a further eight beats were acquired and averaged. The relative change in SBP was then calculated compared to AAI pacing. AAI pacing was then restored and a further eight beats were averaged. This was repeated a minimum of a further three times so that a total of at least eight transitions between AAI and DDD pacing were acquired (Figure 1). The mean relative change for heart rate was calculated from a minimum of eight transitions. This was repeated at each tested AVD. All hemodynamic measurements are subject to variability, which occurs due to respiration, change in sympathetic tone as well as inevitable spontaneous variability inherent to any biological measurement. We have previously shown the importance of performing multiple repeated measurements between a reference setting and a tested setting, in order to prevent spontaneous fluctuations being falsely analyzed as a response to pacing therapy [13]. This approach results in highly reproducible measurements with a high signal‐to‐noise ratio. We measured signal‐to‐noise ratio as the ratio of the peak hemodynamic improvements to the standard error (when data have been fitted to a quadratic curve). Therefore, a larger ratio represents poorer signal‐to‐noise ratio.

The peak SBP response and its confidence intervals were calculated by fitting a quadratic curve to the data from each set of tested AVDs (Figure 1). Analysis of hemodynamic data was automated using Python (Python Software Foundation) so that few user inputs were required and robust regression methods were used so that outliers did not need to be manually removed.

**FIGURE 2:**
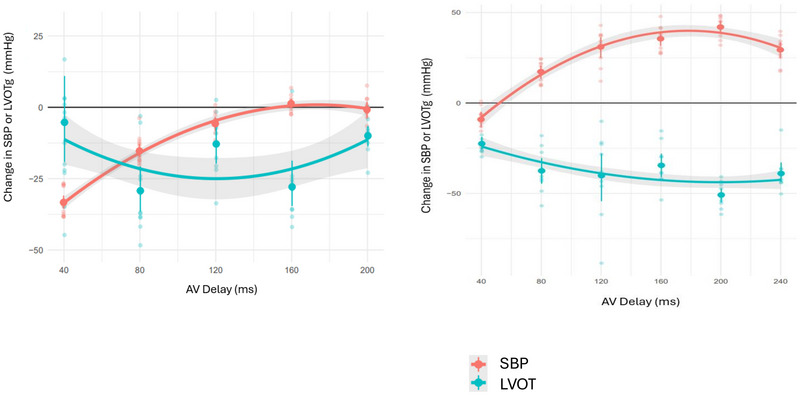
Analysis of hemodynamic and echocardiographic data. For each transition between AAI and DDD pacing (Figure 1), individual heart beat systolic blood pressure (SBP) and left ventricular outflow tract gradient (LVOTg) data points are averaged over eight beats immediately preceding and following each transition. The differences between these averages produce a single ∆SBP and ∆LVOTg data point for each transition, thus 10 data points per atrioventricular delay (AVD) from five alternations. These data are fitted to quadratic to produce narrow confidence intervals for the relationship between AVD and ∆SBP (red)/∆LVOTg (cyan). Two patients’ data are displayed. For the patient on the left, the maximum increase in ∆SBP (the peak of the hemodynamic [∆SBP] curve) is +0.88 mmHg ± standard error 0.84 at AVD 174 ms. At the hemodynamically optimal AVD, right ventricular pacing (RVP) preserves cardiac output, without incrementing or decreasing it. At this AVD, LVOTg is substantially reduced (−18.8 mmHg ± 3.34). At any other AVD, LVOTg is reduced, but RVP reduces SBP compared to baseline. For the patient on the right, the maximum increase in ∆SBP is +39.93 mmHg ± 1.60 at AVD 178 ms. At the hemodynamically optimal AVD, RVP increases cardiac output. At this AVD, LVOTg is substantially reduced (by −43.44 mmHg ± 1.65). If a suboptimal AVD is selected, SBP could be reduced by as much as 30 mmHg by RVP. Most described strategies for AVD selection would identify a suboptimal AVD. Even AVD selection strategies targeting optimal hemodynamics could select suboptimal delay when applied with low‐precision techniques. [Colour figure can be viewed at wileyonlinelibrary.com]

While the above hemodynamic protocol was being performed, continuous beat‐by‐beat recording of continuous wave Doppler through the LVOT was performed. This was carried out by an experienced echocardiographer positioning a probe at the left ventricular apex in five‐chamber view. The Doppler cursor was positioned at the LVOT. Continuous screen capture was performed to ensure beat‐by‐beat data were recorded. The captured screen footage then underwent automated contrast detection to extract the Doppler velocity trace. Peaks were detected (automated) to identify the peak velocity (m/s), which was transformed to gradients (mmHg). We then applied the same high‐precision protocol to these peak gradients (averaging beats pre and post transition multiple times at multiple AVD and fitted to quadratic curves) to ensure the same beats were utilized for both hemodynamic and echocardiographic assessment. This novel, automated, high‐precision assessment of LVOTg response to pacing allows reproducible, unbiased, narrow confidence‐interval assessment (Figure 2). At least 500 data points are used to determine the optimum hemodynamic response and optimum echocardiographic response for each patient at each heart rate.

### 2.3 AVD Selection Strategy Comparison

The optimal hemodynamic response to pacing was recorded at the peak of the parabolic relationship between AVD and ∆SBP. The AVD that produced this optimal hemodynamic response was recorded. The optimal “programmed” AVD was also recorded: the tested AVD producing the highest ∆SBP (as opposed to the peak of the AVD parabola that is typically not a tested or directly programmable AVD). Several other AVD selection strategies that have been applied to identify the appropriate AVD for clinical use were also applied to these data:

- The AVD that produced largest reduction in LVOTg (using high‐precision LVOTg assessment) [14, 15].
- Ultrashort AVD (40–80 ms; 40 ms where this resulted in capture, otherwise 80 ms) [16]
- The longest AVD that entirely captures ventricular myocardium (the longest AVD where fusion or pseudocapture is not observed) [17, 18]
- The nearest tested AVD (in 40‐ms increments) to the hemodynamic optimum AVD (a continuous measure in ms). This also represents a practically programmable AVD.

### 2.4 Statistical Analysis

Baseline characteristics were tabulated and summarized with appropriate statistics. For each patient, an optimal AV‐delay curve was constructed for both the change in beat‐by‐beat blood pressure and at LVOT gradient. From this ordinary least‐squares regression (which included the AV delay as a predictor up to a quadratic polynomial), values for individual AV delays could be predicted along with their associated confidence intervals. From this, the AV delay that produces the greatest response, or the response at any AV delay can be derived. For descriptive statistics and comparisons of the response at specified AV delays or AV delay selection strategies, a group mean and confidence interval based on a one‐sample or paired *t*‐test was used as appropriate. For the signal‐to‐noise ratios, the beat‐by‐beat variability and the variability associated with each “alternation” were calculated from an individual patient, with the beat‐by‐beat variance averaged across multiple alternations. From this, an illustration of their relative importance and capturing multiple repetitions was performed by summating the variances and reducing the variances in proportion to the number of repetitions. Statistical analyses were performed using the statistical environment ‘R’ with the ‘ggplot2’ visualization package [19].

## 3 Results

Twenty two patients were recruited meeting eligibility criteria. Baseline characteristics of these patients are shown in Table 1. Of these 22 patients, 20 patients were studied at 10 bpm above sinus rate. Twenty patients were studied at higher pacing rate (100 bpm in 19 of these, 110 in the remaining patient). In 18 patients, both sinus rate and higher rate were studied.

**TABLE 1:** Baseline characteristics.

Parameter	Value
Age, years	52 ± 13.4 (30–76)
Male	13 (59%)
Ejection fraction, %	60 ± 4.6 (55–70)
Resting LVOTg (mmHg)	54 ± 46 (4–211)
E/A	1.22 ± 0.6 (0.5–2.4)
E/E’	15.8 ± 6.7 (7–28)
PR interval (ms)	172.5 ± 41 (93–261)
QRS duration (ms)	115.8 ± 20.1 (90–166)
NYHA functional class	2.18 ± 0.5 (1–3)
I	1 (5%)
II	16 (73%)
III	5 (22%)
IV	0 (0%)
Transvenous defibrillator	21 (95%)
Permanent pacemaker	1 (5%)
Bisoprolol	18 (82%)
Disopyramide	10 (45%)
Calcium channel blocker	1 (5%)
Cardiac myosin inhibitor	0 (0%)
Anticoagulation	4 (18%)
Hypertension	6 (27%)
Stroke	0
Atrial fibrillation	4 (18%)
Diabetes mellitus	1 (5%)
Ischemic heart disease	3 (14%)
Myectomy	0
Alcohol ablation	0

*Note*: Values are mean ± standard deviation (range) or *n* (%).

Abbreviations: LVOTg, left ventricular outflow tract gradient; NYHA, New York Heart Association.

### 3.1 Hemodynamic and Echocardiographic Responses at Resting Heart Rate

**FIGURE 3:**
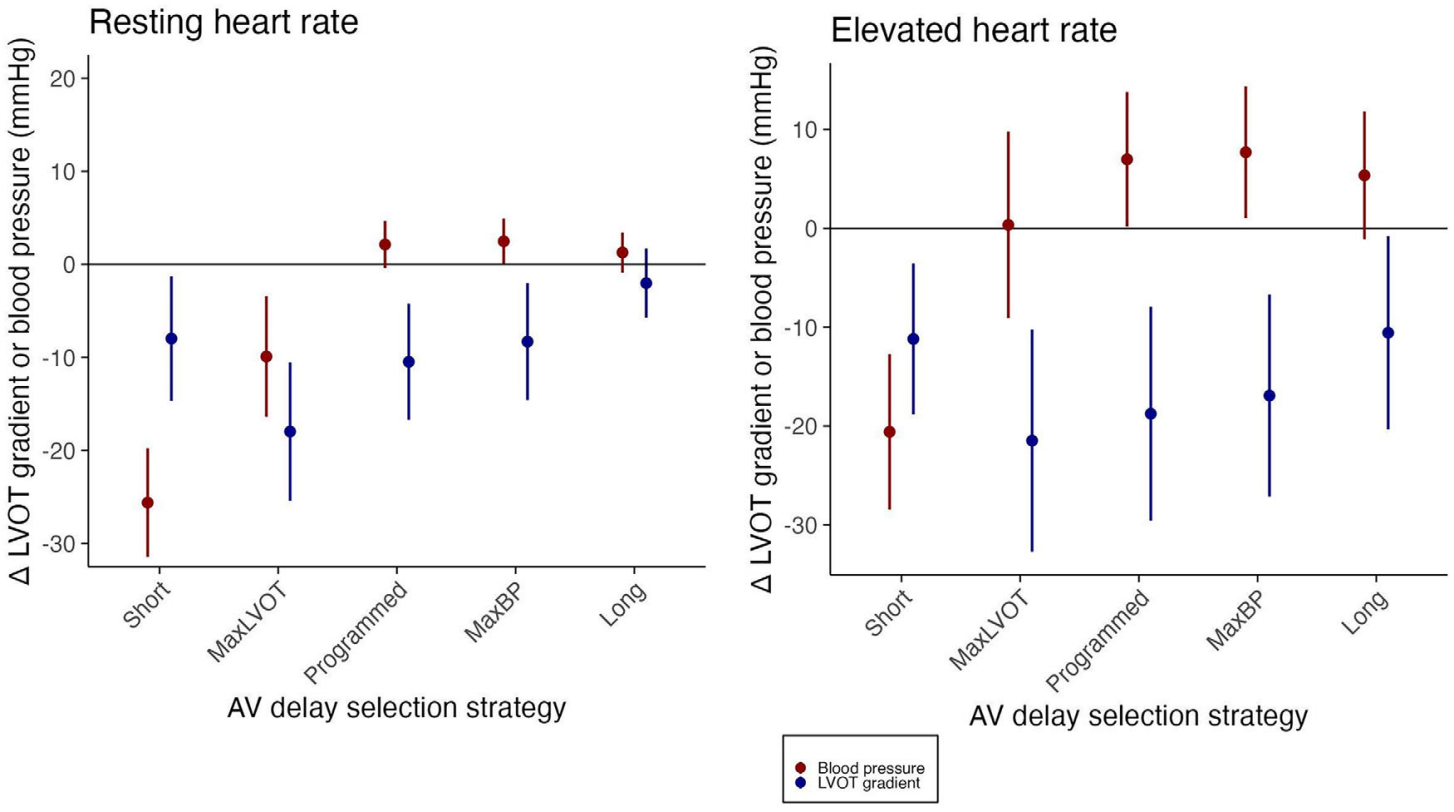
Summary of hemodynamic and echocardiographic effects of different atrioventricular delay selection strategies. ∆SBP and ∆LVOTg with standard errors for all studied patients are shown for pacing close to sinus rate and pacing at an elevated rate (100 bpm). Five described AVD selection strategies are shown in order of increasing AVD: (i) Short—ultrashort AVD of 40–80 ms, (ii) MaxLVOT—AVD producing the largest reduction in LVOTg, (iii) Programmed AVD (iv) MaxBP—AVD producing the maximum ∆SBP, (v) Long—Longest AVD that does not allow intrinsic conduction. Although most strategies reduce LVOTg (blue), only selecting the hemodynamically optimal AVD (MaxBP) produces a statistically significant increase in cardiac output alongside a drop in LVOTg. Ultrashort AVDs systematically impair cardiac output. Other strategies produce suboptimal cardiac output increment. [Colour figure can be viewed at wileyonlinelibrary.com]

**FIGURE 4:**
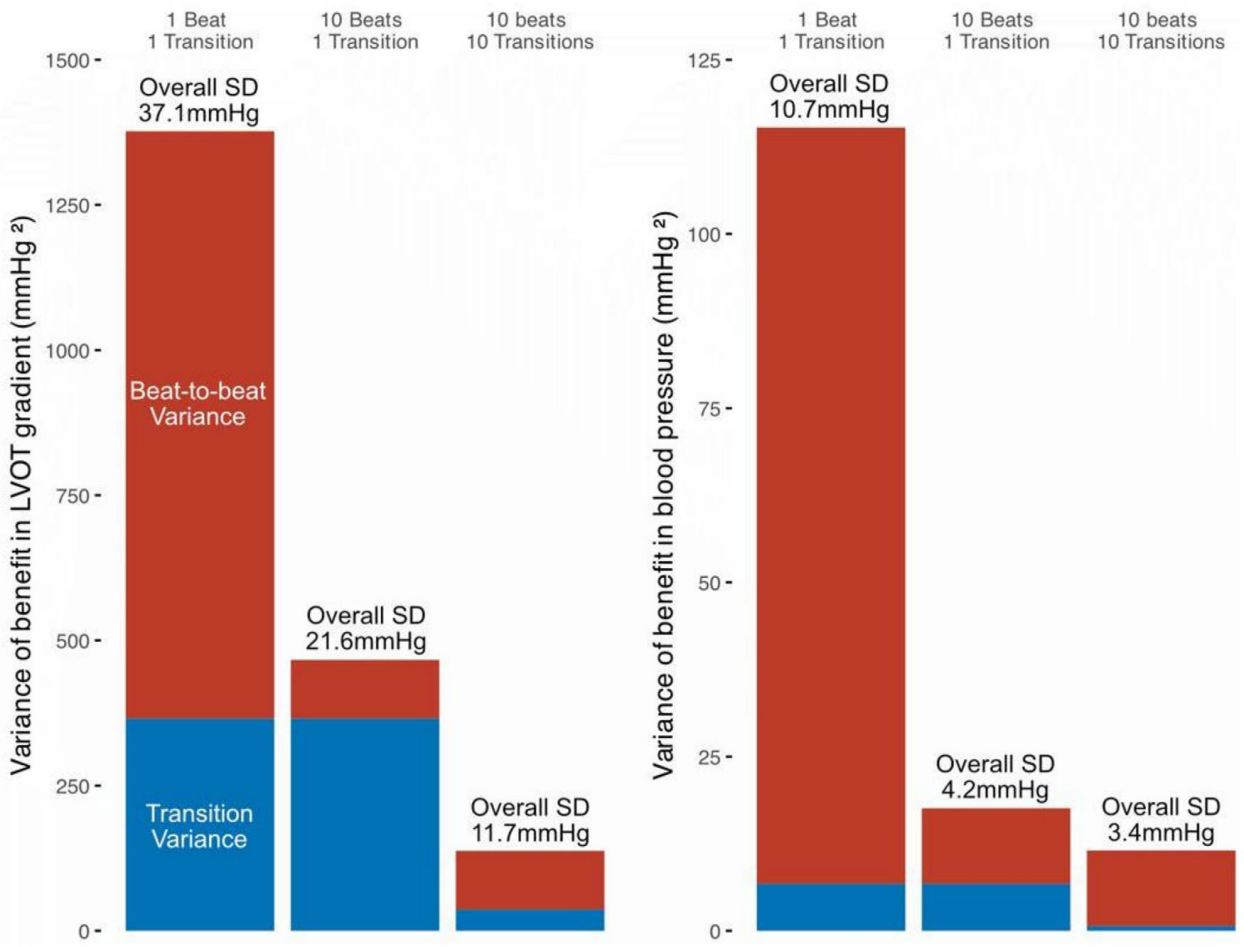
High‐precision methodology improves signal‐to‐noise ratio. Alternation variance (blue) is the standard deviation (SD) of ∆LVOTg (left) or ∆SBP (right) data points for transitions between AAI and DDD pacing. Beat‐to‐beat variance (red) is the SD of BP and LVOTg data points for studied heart beats. Alternation and beat‐to‐beat variance are added to form a single bar of overall variance. From left to right on each chart, sequentially increased precision is applied. On the left of each chart, variance for studying single heartbeat data points for single transitions from AAI to DDD is shown. The middle bar of each chart shows increasing the number of transitions between AAI and DDD to 10, but continuing to study just one heart beat before and after each transition. The right bar of each chart shows the effect of 10 transitions while averaging 10 heart beats before and after each transition. Studying multiple transitions reduces transition variance (red). Averaging multiple heart beats reduces beat‐to‐beat variance (blue). In this way, high‐precision, multiple alternation methodology produces narrow confidence intervals for estimates of ∆SBP and ∆LVOTg. [Colour figure can be viewed at wileyonlinelibrary.com]

The mean resting paced rate was 75 bpm (range 70–90). At just above resting heart rate, the mean peak change in SBP from AAI to DDD was +2.47 mmHg (95% CI 0.19–4.76, *p* = 0.04) and the mean AVD at which this was achieved was 173.2 ms. The mean change in LVOTg at this AVD was −8.31 mmHg (95% CI −2.43 to −14.18, *p* < 0.001). The median optimal programmable AVD was 160 ms (range 120–240). At optimal programmed AVD, SBP change was +2.13 mmHg (95% CI −0.24 to 4.51, *p* = 0.09) and LVOTg change was −10.47 (−4.63 to −16.32, *p* < 0.001). In 35% of patients (7/20), a hemodynamic benefit was observed with the patient's lower limit of the confidence interval for RVP ∆SBP being greater than 0 mmHg. In 60% (12/20), cardiac output was preserved, but not improved by RVP: the confidence interval spanned 0 mmHg. Thus, in 95% (19/20), cardiac output was either preserved or increased by RVP. Selecting the shortest AVD (AVD 40 ms in 19/20, 80 ms in 1/20) produced a mean SBP change of −25.60 mmHg (95% CI −20.15 to −31.1, *p* < 0.001) and LVOTg change of −7.98 mmHg (95% CI −1.71 to −14.24, *p* < 0.001). Selecting the longest capturing AVD (mean 193.5) produced a mean SBP change of +1.27 mmHg (95% CI −0.74 to +3.3 mmHg, *p* = 0.22) and LVOTg change of −2.02 mmHg (95% CI +1.46 to −5.49, *p* = 0.27). At the AVD producing largest LVOTg reduction (mean 102 ms), mean change in SBP was −9.91 mmHg (95% CI −3.84 to −15.98, *p* = 0.004) and mean LVOTg change was −17.97 mmHg (−11.00 to −24.93 mmHg, *p* < 0.001). These results are summarized in Figure 3. Only when selecting hemodynamically optimal AVDs was a statistically significant pacing‐induced improvement in SBP observed. The signal‐to‐noise ratio of ∆SBP was −1.41 − 18.15:1 and ∆LVOTg was −23.98 − 11.13:1 (Figure 4).

### 3.2 Hemodynamic and Echocardiographic Responses at Elevated Heart Rate

At 100 bpm, the mean peak change in SBP from AAI to DDD was +7.70 mmHg (95% CI +1.45 to +13.94, *p* = 0.02) and the mean AVD at which this was achieved was 164.5 ms. The mean LVOTg reduction at this AVD was −16.91 mmHg (95% CI −7.34 to −26.49, *p* < 0.001). The median optimal programmable AVD was 160 ms (range 120–200). At optimal programmed AVD, SBP change was +6.98 mmHg (95% CI +0.61 to +13.36, *p* = 0.04) and LVOTg change was −18.75 (−8.62 to −28.88, *p* = 0.002). In 50% of patients (10/20), a hemodynamic benefit was observed with the patient's lower limit of the confidence interval for RVP ∆SBP being greater than 0 mmHg. In 40% (8/20), cardiac output was preserved, but not improved by RVP: the confidence interval spanned 0 mmHg. Thus, in 95% (18/20), cardiac output was either preserved or increased by RVP at 100 bpm. Selecting the shortest AVD (AVD 40 ms in 16/20, 80 ms in 4/20) produced a mean SBP change of −20.60 mmHg (95% CI −13.21 to −27.95, *p* < 0.001) and LVOTg change of −11.20 mmHg (95% CI −4.04 to −18.33, *p* = 0.006). Selecting the longest capturing AVD (mean 194) produced a mean SBP change of +5.36 mmHg (95% CI −0.68 to +11.41 mmHg, *p* = 0.09) and LVOTg change of −10.51 mmHg (95% CI −1.40 to −19.71, *p* = 0.04). At the AVD producing largest LVOTg reduction (mean 123 ms), mean change in SBP was +0.34 mmHg (95% CI −8.48 to 9.18, *p* = 0.93) and mean LVOTg change was −21.47 mmHg (−10.97 to −31.99 mmHg, *p* < 0.001). These results are summarized in Figure 3. Only by selecting hemodynamically optimal AVDs, was statistically significant pacing‐induced improvement in SBP observed. The signal‐to‐noise ratio of ∆SBP was −2.76 − 24.80:1 and ∆LVOTg was −27.27 − 0.20:1 (Figure 4). The signal‐to‐noise for ∆SBP and ∆LVOTg at elevated heart rate is depicted in Figure S1. Peak ∆SBP rise and ∆LVOTg reduction at 100 bpm were numerically higher than at just above sinus rate.

## 4 Discussion

In this study we have, for the first time, quantified with high precision the hemodynamic and echocardiographic effects of RVP on LVOTO in patients with oHCM. Our methodology utilizes multiple measurements alternating between reference and test states to achieve reproducible results and allowed us to quantify the signal‐to‐noise ratio of blood pressure and continuous wave LVOTg Doppler responses. This is the first time this noise and bias resistant approach has been applied to echocardiographic data. There were several important findings:

- High‐precision measurement methodology allows reproducible assessment of the direction and magnitude of hemodynamic and echocardiographic responses to RVP in oHCM, with narrow confidence intervals.
- RVP can reduce LVOTg while preserving or improving cardiac output, but AVD is a key modifier of the hemodynamic and LVOTg response to RVP.
- Ultrashort AVDs reduce LVOTg at the cost of worsened cardiac output and the AVD that minimizes LVOTg is not necessarily the optimal AVD for overall cardiac function.
- RVP produces larger reductions in LVOTg and larger increases in cardiac output at higher heart rates.

### 4.1 Right Ventricular Pacing in HCM

The mechanistic potential for RVP to improve LVOTO in oHCM was first recognized in the 1980s [20]. In oHCM, the combination of LVOT narrowing, due to asymmetric septal hypertrophy, and excessively forceful LV contraction, due to hypertrophied myocardium, results in turbulent flow in the LVOT, which drags the anterior mitral valve leaflet toward the septum, through the Bernoulli principle, to further narrow the LVOT and obstruct flow [21]. This dynamic LVOT obstruction is a key driver of symptoms and prognosis in HCM. RVP produces a dyssynchronous ventricular activation pattern with lateral wall contraction delayed relative to the septum. It followed that by disrupting the coordinated, synchronous ventricular contraction produced by native activation via the His‐Purkinje conduction system, RVP could prevent LVOT constriction throughout systole since, at any given systolic timepoint, only part of the LV would be contracting. Contemporaneous studies of RVP in heart failure demonstrated the risk of dyssynchrony‐induced LV pump impairment [22]. In oHCM, this effect is both therapeutic and potentially harmful: RVP‐induced dyssynchrony reduces the hypercontractile LV driving force on blood into the LVOT to decrease flow turbulence, but may also reduce overall cardiac output through ventricular impairment. Our findings demonstrate that in most patients it is possible for the trade‐off between these effects to favor a net hemodynamic benefit for RVP with LVOTg reduction. The 32% reduction in LVOTg we observed is similar to that observed in randomized trials (35%). Our study clearly shows that AVD is a crucial modifier of this balance between hemodynamic harm and benefit.

### 4.2 AVD Selection Strategies

Dual chamber pacing allows control of AVD in AV‐sequential pacing modes, which introduces a third factor (in addition to dyssynchrony and myocardial performance). Early studies of RVP in oHCM favored ultrashort AVDs, which seemed to produce very large reductions in LVOTg [16, 23]. Our findings demonstrate that this is not a physiologically beneficial way of reducing LVOTg. Overly short AVDs curtail diastolic LV filling to impair subsequent systolic ejection. The resultant reduced LV driving force produces less turbulent LVOT flow but, as our data show, it does this by also considerably impairing cardiac output. As this phenomenon was appreciated, longer AVDs were implemented. It was recognized that very long AVDs would allow fusion with intrinsic AV conduction, which would attenuate the potential benefits of induced dyssynchrony. A common AVD selection strategy was to identify the longest AVD that fully captured the ventricle (without fusion or pseudocapture) [24]. As our findings show, overly long AVDs may not be the most beneficial way of reducing LVOTg through RVP, although the difference is not as stark as compared with ultrashort AVDs. Long AVDs allow presystolic mitral regurgitation, which also underloads the LV, impairing pump function—mitigating cardiac output incrementation. We also found that LVOT reduction does not occur consistently at these longer AVDs.

In order to balance dyssynchrony‐induced LVOTO improvement, dyssynchrony‐induced LV pump dysfunction, and varying AVD effects on LV filling, later studies involved recording aortic pressure along with LVOTg at a range of AVDs to find the AVD that preserves or improves aortic pressure while reducing LVOTg. This was used in the three double‐blinded randomized trials of RVP in oHCM [2, 8, 25]. Our data show that this strategy is physiologically sound, but the measurement methodology must be precise. By manually recording single or low‐multiple measurements of aortic pressure and LVOTg at different AVDs and picking the apparently optimal AVD, there is considerable scope for bias and noise to allow a suboptimal AVD to be incorrectly selected.

Unblinded manual measurements are prone to biased reporting. Echocardiographic interpretation, including Doppler tracing, is particularly susceptible to this, but BP measurements are also affected. User bias is introduced through multiple means such as beat selection and subjectivity of manual spectral tracing. Our high‐precision method avoids this by applying automated beat selection and automated measurements of BP and continuous wave Doppler.

There is natural, spontaneous variation in all biological measurements. Our data show that single measures of BP and echo‐derived LVOTg are subject to very poor signal‐to‐noise ratios (Figure 4). Variation can be exogenous in origin (respiratory cycle, autonomic changes) or inherent to signal and measurement apparatus. By alternating between reference and test states, baseline drift is accounted for in our method and averaging multiple measurements improves reproducibility. This allowed us to precisely determine at which AVDs cardiac output and LVOTO were impaired or improved and to what extent. By producing narrow confidence intervals, we provide estimates for optimum AVD that are reproducible within patients. This is the first time this method has been applied to echocardiographic data and the first time for blood pressure in the context of HCM. The technique's precision has allowed us to demonstrate gross differences between the hemodynamics of optimal AVD and very short AVD and between the LVOTg reductions of optimal AVD and very long AVDs, but subtle differentiations are also made possible. It is evident across patients and within patients that the AVD that produces the lowest LVOTg is not the same as the AVD that maximizes cardiac output, although this difference is smaller than comparisons with ultrashort delays. This suggests that the challenge in selecting an AVD for an individual patient is one of balancing LVOTg reduction and overall cardiac function through the interplay of physiological factors, rather than a pure “optimization” challenge.

### 4.3 Clinical Trials of RVP for HCM

Observational studies of RVP in HCM were initially promising, associating large improvements in LVOTg and symptoms with RVP. Many of these studies utilized AVDs that our findings have shown to be hemodynamically deleterious. Our meta‐analysis [26] revealed that blinded RCTs confirmed a true reduction in LVOTg from RVP, but failed to demonstrate consistent symptomatic improvement, suggesting that unintended bias was responsible for much of the apparent symptomatic benefit seen in the observational studies. As a result, RVP for oHCM carries weak guideline support and is not a widely used therapy. The mismatch between LVOTg reduction and symptom improvement in RCTs may have been due to a low‐precision method for AVD selection. It is clear that even a 40 ms change in AVD from hemodynamic optimum could result in pacing being hemodynamically harmful rather than beneficial despite lowering LVOTg. However, most patients we studied did demonstrate an AVD that either preserved or improved cardiac output while reducing LVOTg. By employing our high‐precision technique to select the most hemodynamically beneficial AVD and applying a high‐resolution symptom measure, we hope to demonstrate that RVP does improve symptoms in oHCM in the EMORI‐HCM randomized, double‐blinded crossover trial (NCT05257772).

### 4.4 Heart Rate Effects

The magnitude of RVP‐induced cardiac output incrementation and LVOTg reduction at optimal AVDs were greater at elevated heart rates compared to heart rates very close to sinus rate. Since exertion is a common precipitant for LVOTO‐related symptoms in oHCM, this may represent both the ability of exertional heart rates to worsen LVOTO due to inadequate filling and hypercontractility and the commensurately higher benefits of pacing in this scenario as a result of this. However, it should be noted that high‐rate atrial pacing is not a perfect surrogate for exertion because atrial pacing does not generally entail other exercise effects such as vasodilatation.

### 4.5 Impact of High‐Precision Estimates of RVP Benefit

If AV‐optimized RVP improves symptoms, this will have immediate impact on clinical care of patients with symptomatic oHCM. Many patients with HCM have dual chamber pacing devices already implanted (pacemakers and defibrillators), which could be hemodynamically optimized and set to provide pacing [27, 28, 29]. Importantly, when defibrillator implant is indicated and clinicians and patients are choosing between transvenous and subcutaneous devices, a confirmed benefit of RVP could influence this decision as only transvenous devices can provide this. When transvenous devices are implanted, the addition of an atrial lead is typically only necessary when atrioventricular block has been observed, but hemodynamically beneficial AV‐sequential RVP could influence this decision, as an atrial lead is required for this. Our method also provides a way of determining which individual patients will gain a net benefit from RVP and to what extent: a high‐precision estimate of the change in cardiac output with pacing, which balances dyssynchrony‐induced LVOT changes, ventricular function, and LV filling through AVD variation. This could be performed using electrophysiological catheters to inform defibrillator decisions.

We found that, at optimal AVD, RVP produces a 2.47 mmHg SBP improvement at resting heart rates. Improvements of this magnitude or smaller have been correlated with symptomatic improvements [4]. Biventricular pacing as cardiac resynchronization therapy produces at 7.8 mmHg improvement in SBP, when measured in the same high precision way. 30% of the mortality and symptom benefits of biventricular pacing is a clinically meaningful benefit if our findings extend to clinical endpoints, which is being assessed in EMORI‐HCM (NCT05257772). The magnitude of SBP improvement is not the same as the LVOT gradient reduction as the former is modulated by LVOTg, AVD‐related filling, and myocardial performance impairment by RVP.

There are other therapies available for LVOT gradient reduction. Some patients are unable or unwilling to undergo interventions (ethanol ablation, myectomy, etc.) and they can be incomplete. Medical therapy can be poorly tolerated and incompletely effective, including the newer myosin inhibitors, which, at present, require intensive echocardiographic follow‐up that has resource utilization implications. As many patients already have dual chamber pacing devices (PPM/ICD) implanted, pacing could be an important therapy alongside or in lieu of these treatments, but only when a net cardiac performance benefit is achieved.

### 4.6 Limitations

Although our findings are statistically significant, we do not yet know if RVP‐induced cardiac output changes correlate with clinically important symptomatic improvement, which will be assessed in the EMORI‐HCM randomized crossover trial, or long‐term outcomes, which will require evaluation in event‐driven trials. Elevating the heart rate by increasing the lower rate limit in DDD mode is not the same as exercise‐induced tachycardia, which is associated with changes in myocardial performance and peripheral vasodilation. AVDs were assessed in atrial pacing (ApVp), as opposed to atrial sensing (AsVp), as the latter is prone to noise from fluctuating sinus rate. It is feasible to perform this assessment for atrial sensing, but atrial pacing results can be approximated to apply to sensed AVD programming by removing 30 ms. In some patients, LVOT was not greatly reduced by RVP and it is possible that almost all of the hemodynamic benefits seen at optimal AVD in these patients were due entirely to shortened AVD (and not from LVOT alleviation). The HOPE‐HF trial [4] and other hemodynamic studies have shown that AV shortening is in itself beneficial in many patients whose intrinsic AVD is long. It is also important to note that in this study, only noninvasive methods have been used. Invasive hemodynamic and LVOTg measurements in addition to our noninvasive SBP assessment would provide more clarity into the exact nature of cardiac output incrementation. Furthermore, our findings are only applicable to patients who fit specific inclusion criteria (dual chamber device, sinus rhythm, narrow intrinsic QRS, and low/no burden of atrial fibrillation). Patients with a narrow QRS duration are likely to have the most to gain by RVP‐induced dyssynchrony. It may, however, be possible that patients with a wider intrinsic QRS could still benefit if RVP produces more dyssynchrony than their intrinsic QRS.

## 5 Conclusion

High‐precision measurement methodology allows reproducible assessment of the direction and magnitude of hemodynamic and echocardiographic responses to RVP in oHCM, with narrow confidence intervals. RVP can reduce LVOTg while preserving or improving cardiac output, but AVD is a key modifier of the hemodynamic and LVOTg response to RVP.

## Disclosure

A.D.A. has received honoraria from Medtronic, Abbott, and Biotronik and travel support from Bayer. F.S. has received travel support from Servier and honoraria from Philips. D.K. has received honoraria and research funding from Abbott and Medtronic and honoraria from Biotronik. Z.I.W. has received honoraria and research funding from Medtronic, Boston Scientific, and Abbott and honoraria from Biotronik. M.T. has received educational support from Medtronic. J.P.H. is a shareholder in MyCardium AI Ltd.

## Supporting information




**Supporting File 1**: pace70024‐sup‐0001‐Appendix.docx.

**Supplementary Fig. 1**: High Precision Methodology Improves Signal‐To‐Noise Ratio at Elevated Heart Rates.

**Supplementary Fig. 2**: Change in Systolic Blood Pressure measured at transition points between AAI and DDD pacing and between DDI and AAI pacing.

**Supplementary Fig. 3**: Change in LVOT Gradient measured at transition points between AAI and DDD pacing and between DDI and AAI pacing.

**Supplementary Fig. 4**: Consort Diagram.
