# Supplementary material for: High‐Precision Hemodynamic and Echocardiographic Assessment of Pacing in Obstructive Hypertrophic Cardiomyopathy
Source: Pacing Clin Electrophysiol. 2025 Aug 21;48(10):1138–47. doi: 10.1111/pace.70024 (PMC12504915; doi:10.1111/pace.70024)
Supplement: Supplementary file 1 — Supporting File 1: pace70024‐sup‐0001‐Appendix.docx. [file PACE-48-1138-s005.docx]

**Supplementary Appendix**

**Contents**

1. **Supplementary Figure 1:** High Precision Methodology Improves Signal-To-Noise Ratio at Elevated Heart Rates.
2. **Supplementary Figure 2:** Change in Systolic Blood Pressure measured at transition points between AAI and DDD pacing and between DDI and AAI pacing.
3. **Supplementary Figure 3:** Change in LVOT Gradient measured at transition points between AAI and DDD pacing and between DDI and AAI pacing.
4. **High Precision Haemodynamic and Echocardiographic Assessment**
5. **Supplementary Figure 4:** Consort Diagram

| 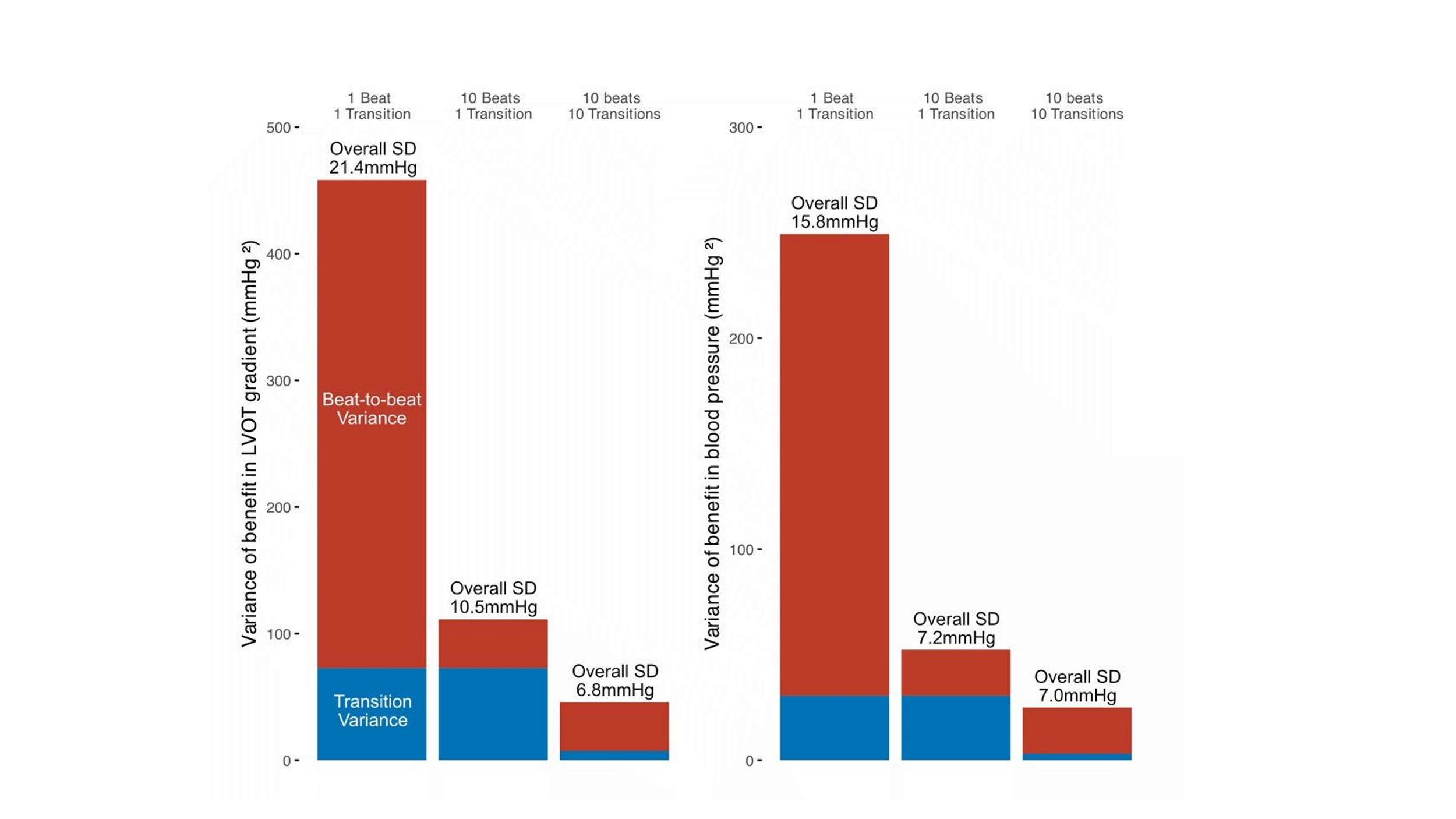 |
| --- |
| **Supplementary Figure 1: High Precision Methodology Improves Signal-To-Noise Ratio At Elevated Heart Rates.**  Signal to noise ratio, as depicted in Figure 4, is shown here for elevated heart rates. Alternation variance (blue) is the standard deviation (SD) of ∆LVOTg (left) or ∆SBP (right) data points for transitions between AAI and DDD pacing. Beat-to-beat variance (red) is the SD of BP and LVOTg data points for studied heartbeats. Alternation and beat-to-beat variance are added to form a single bar of overall variance. From left to right on each chart, sequentially increased precision is applied. On the left of each chart, variance for studying single heart beat data points for single transitions from AAI to DDD are shown. The middle bar of each chart shows increasing the number of transitions between AAI and DDD to ten but continuing to study just one heartbeat before and after each transition. The right bar of each chart shows the effect of ten transitions while averaging ten heartbeats before and after each transition. Studying multiple transitions reduces transition variance (red). Averaging multiple heartbeats reduces beat-to-beat variance (blue). In this way, high-precision, multiple-alternation methodology produces narrow confidence intervals for estimates of ∆SBP and ∆LVOTg.  ∆LVOTg – Change in Left Ventricular Outflow Tract Gradient; ∆SBP – Change in Systolic Blood Pressure |

| 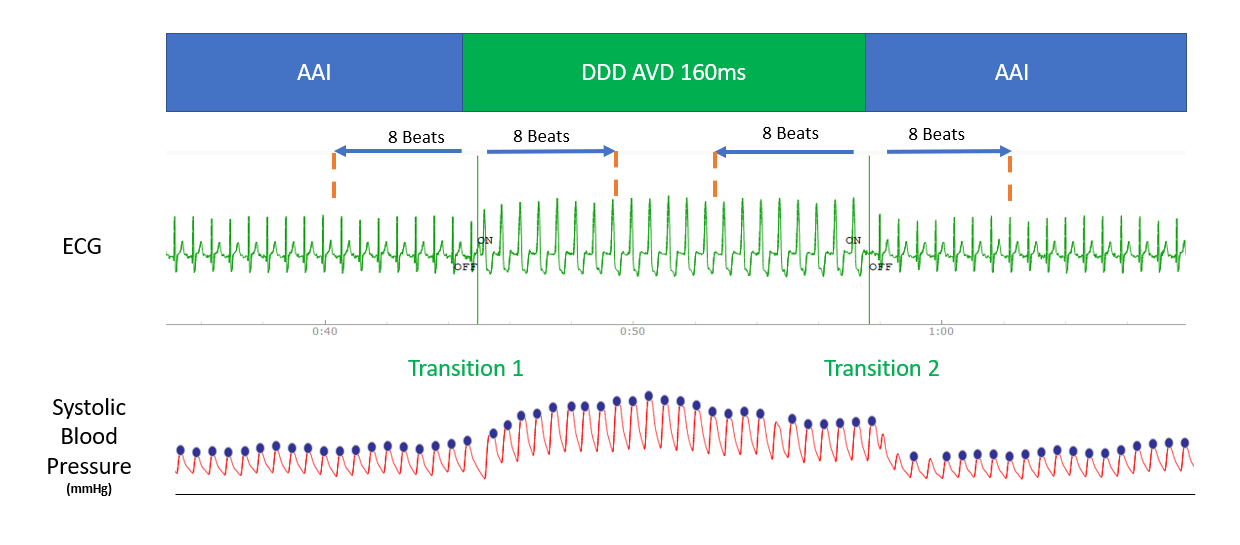 |
| --- |
| **Supplementary Figure 2: Change in Systolic Blood Pressure measured at transition points between AAI and DDD pacing and between DDI and AAI pacing.** Transition 1 uses the 8 beats of AAI pacing before a transition is made to DDD pacing. The average SBP of the 8 beats before and after the transition is used to give a ΔBP measurement for Transition 1. At Transition 2, the average SBP of 8 beats before and after the transition is made from DDD to AAI pacing is used to give a ΔSBP measurement for Transition 2.  ∆SBP – Change in Systolic Blood Pressure |

| 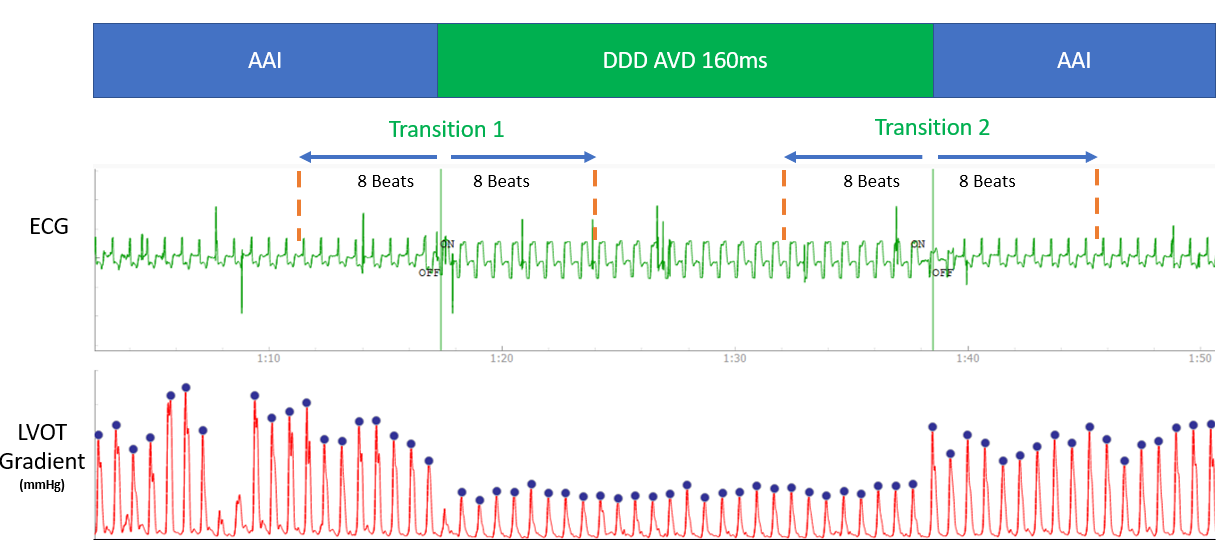 |
| --- |
| **Supplementary Figure 3: Change in LVOTg measured at transition points between AAI and DDD pacing and between DDI and AAI pacing.** Transition 1 uses the 8 beats of AAI pacing before a transition is made to DDD pacing. The average LVOTg of the 8 beats before and after the transition is used to give a ΔLVOTg measurement for Transition 1. At Transition 2, the average LVOTg of 8 beats before and after the transition is made from DDD to AAI pacing is used to give a ΔLVOTg measurement for Transition 2.  ∆LVOTg – Change in Left Ventricular Outflow Tract Gradient |

**High Precision Haemodynamic and Echocardiographic Assessment**

Waveforms of non-invasive beat-by-beat blood pressure (BP), continuous wave doppler through the left ventricular outflow tract and 3-lead ECG were continuously recorded during the protocol. A range of AVDs were tested, from 40ms until pure intrinsic conduction occurred (in 40ms increments). Pacing was alternated between AAI pacing, as the reference state, and atrioventricular sequential RV pacing (DDD pacing), as the test state, multiple times for a given AVD.

For a single AVD the haemodynamic assessment was performed as follows. Eight beats of blood pressure data were acquired prior to a transition from AAI to DDD (or DDD to AAI). The systolic pressure (SBP) of each beat was detecting using a custom peak detection algorithm. The average SBP of the eight beats of pacing immediately before a transition was automatically calculated as the pre-transition SBP. Following the transition, the next immediate eight beats were acquired and averaged to calculate the post-transition SBP. The change in SBP (∆SBP) from pre to post transition was calculated as post-transition SBP minus pre-transition SBP. The relative change for DDD to AAI transitions was calculated as pre-transition SBP minus post-transition SBP so that all transition SBP changes were in the same direction. After five alternations (10 transitions), 10 ΔSBPs were determined for each AVD producing 10 data points at each AVD. This allows calculation of the mean ∆SBP from reference to test state for a given AVD and a confidence interval.

For each AVD, this process was repeated at each tested AVD. The reference state remains constant (AAI) but the test state as DDD changes AVD. This produces 10 ∆SBP data points per AVD, therefore up to 80 data points (for AVDs from 40ms to 320ms creating 1,280 beats worth of data) can be plotted on a graph of ∆SBP (y axis) against AVD (x axis).

From multiple studies (1-4) and the expected underfilling at short AVDs and overfilling alongside fusion at long AVDs, it was expected that the relationship of ∆SBP and AVD would be well fitted to a quadratic equation. By fitting the data to a quadratic, a quadratic line could be produced that represents the overall relationship between ∆SBP and AVD, with very narrow confidence intervals produced by this quadratic assumption. This allows estimation of the optimal haemodynamic AVD and the haemodynamic effect of this AVD, as well as estimation of the haemodynamic effect at any AVD, which we applied in this study.

Echo data was analysed in a similar way. The doppler cursor was positioned at the LVOT. Continuous screen capture was performed to ensure beat-by-beat data was recorded. The captured screen footage then underwent automated contrast detection to extract the doppler velocity trace. Peaks were detected (automated) to identify the peak velocity (m/s), which was transformed to gradients (mmHg). We then applied the same high-precision protocol to these peak LVOT gradients (averaging beats pre and post transition multiple times at multiple AVD and fitted to quadratic curves) to ensure the same beats were utilised for both haemodynamic and echocardiographic assessment.

**Supplementary Figure 4:** Consort Diagram

**
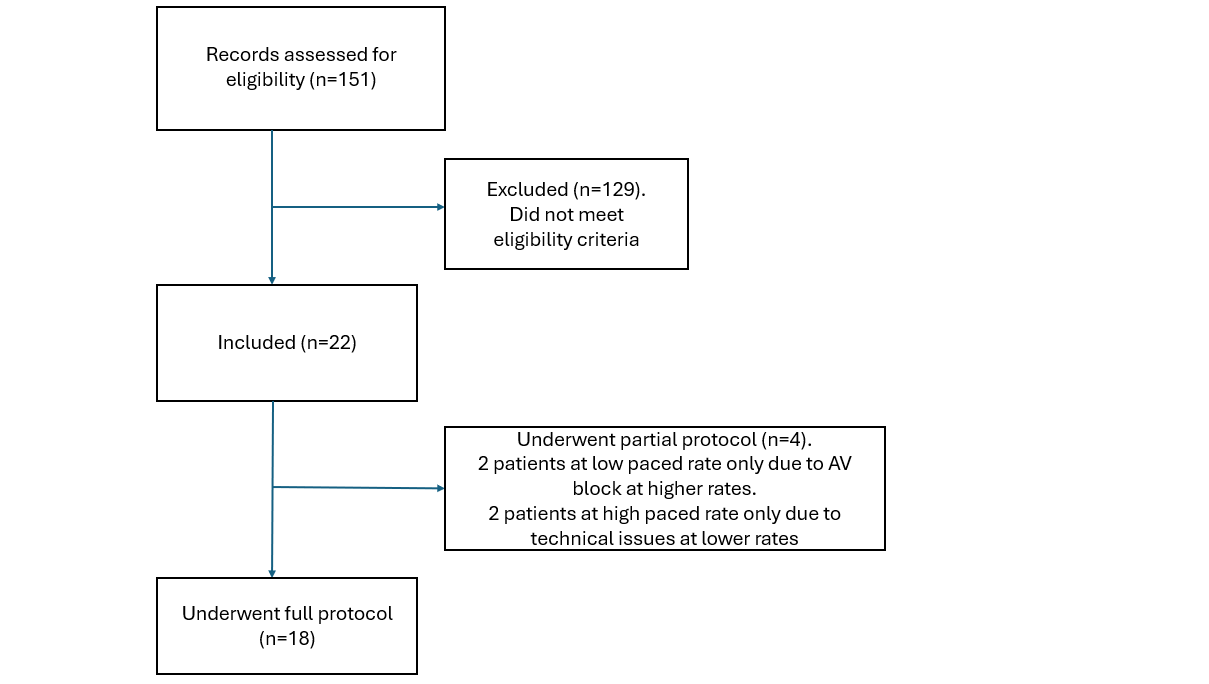
**

**Consort Diagram**: Showing the number of patients screened and how many were included and excluded.

**References**

1. Sharp AJ, Sohaib SMA, Shun-Shin MJ, Pabari P, Willson K, Rajkumar C, et al. Improving haemodynamic optimization of cardiac resynchronization therapy for heart failure. Physiol Meas. 2019;40(4):04nt1.

2. Miyazawa AA, Francis DP, Whinnett ZI. Basic Principles of Hemodynamics in Pacing. Card Electrophysiol Clin. 2022;14(2):133-40.

3. Whinnett ZI, Davies JE, Willson K, Chow AW, Foale RA, Davies DW, et al. Determination of optimal atrioventricular delay for cardiac resynchronization therapy using acute non-invasive blood pressure. Europace. 2006;8(5):358-66.

4. Whinnett ZI, Sohaib SMA, Mason M, Duncan E, Tanner M, Lefroy D, et al. Multicenter Randomized Controlled Crossover Trial Comparing Hemodynamic Optimization Against Echocardiographic Optimization of AV and VV Delay of Cardiac Resynchronization Therapy: The BRAVO Trial. JACC Cardiovasc Imaging. 2019;12(8 Pt 1):1407-16.
