# Supplementary figures and images for: High‐Precision Hemodynamic and Echocardiographic Assessment of Pacing in Obstructive Hypertrophic Cardiomyopathy
Source: Pacing Clin Electrophysiol. 2025 Aug 21;48(10):1138–47. doi: 10.1111/pace.70024 (PMC12504915; doi:10.1111/pace.70024)

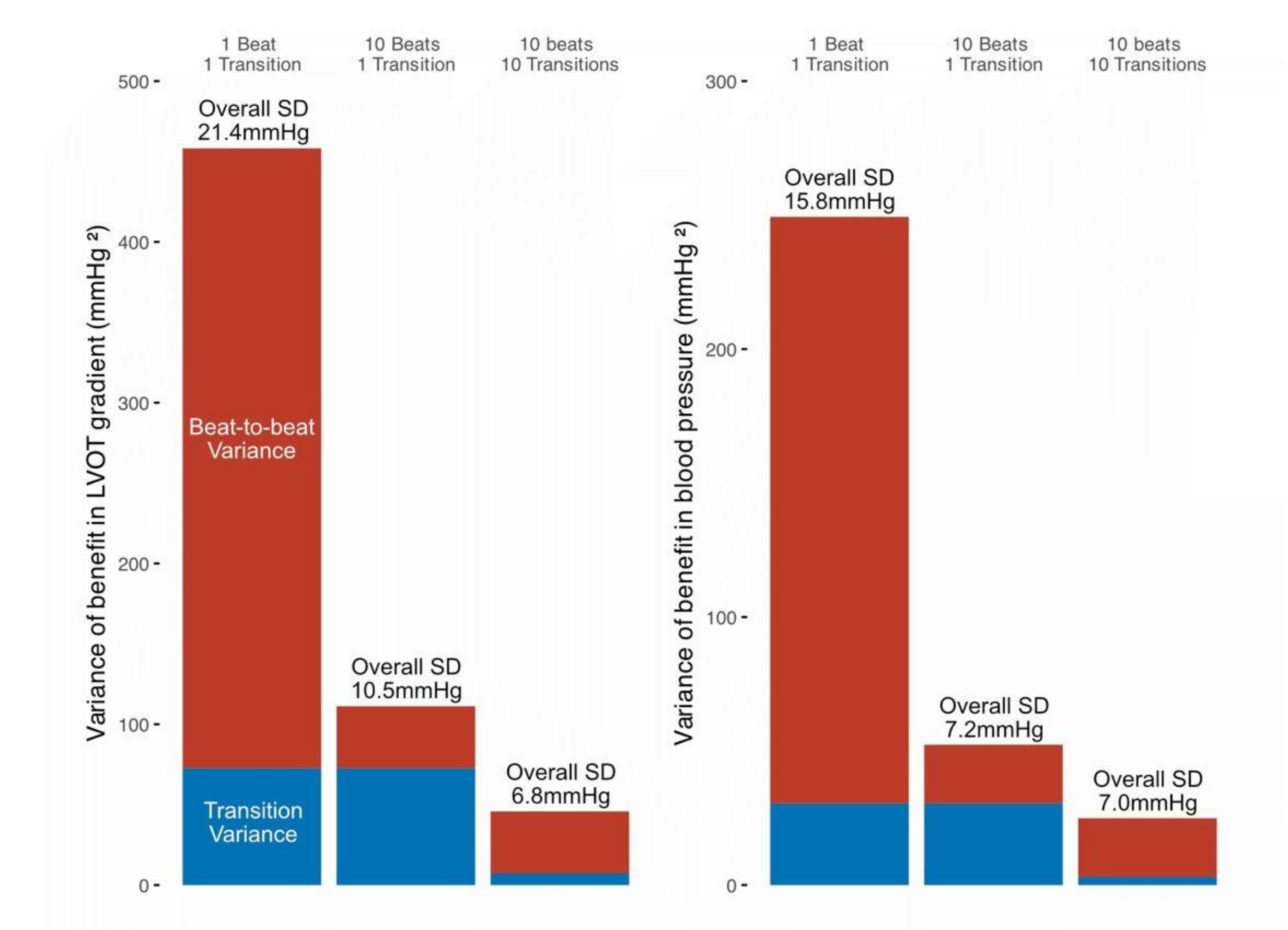

Supplement: Supplementary file 2 — Supplementary Fig. 1: High Precision Methodology Improves Signal‐To‐Noise Ratio at Elevated Heart Rates. [file PACE-48-1138-s002.tif]

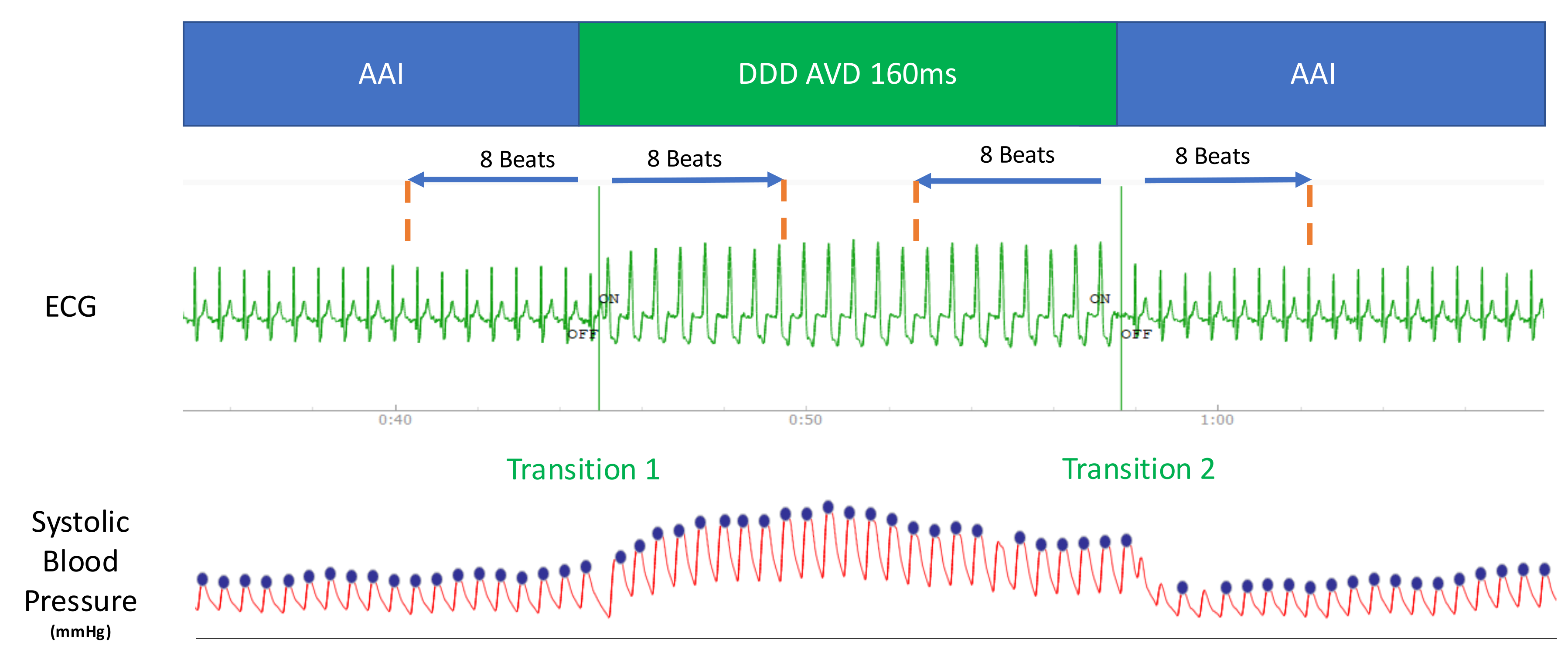

Supplement: Supplementary file 3 — Supplementary Fig. 2: Change in Systolic Blood Pressure measured at transition points between AAI and DDD pacing and between DDI and AAI pacing. [file PACE-48-1138-s003.tif]

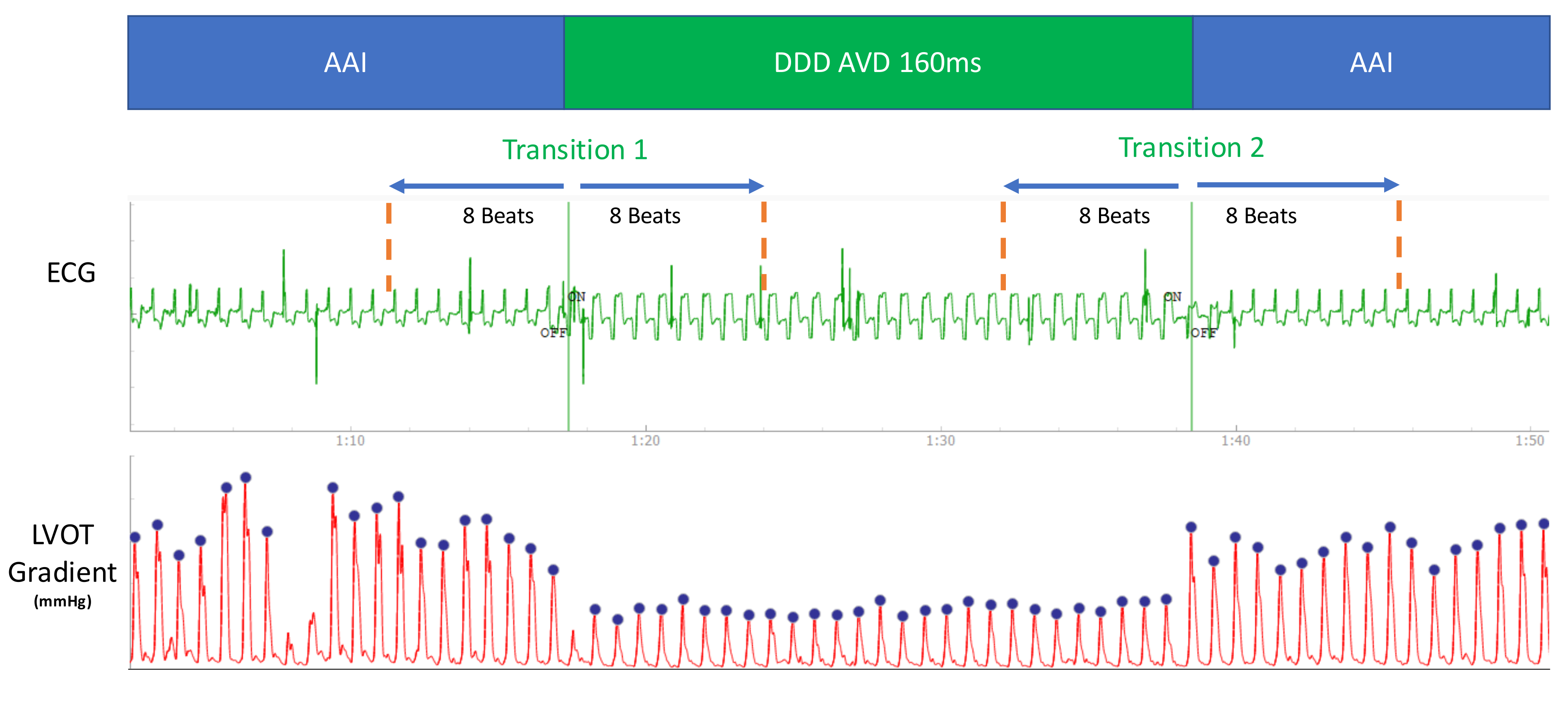

Supplement: Supplementary file 4 — Supplementary Fig. 3: Change in LVOT Gradient measured at transition points between AAI and DDD pacing and between DDI and AAI pacing. [file PACE-48-1138-s001.tif]

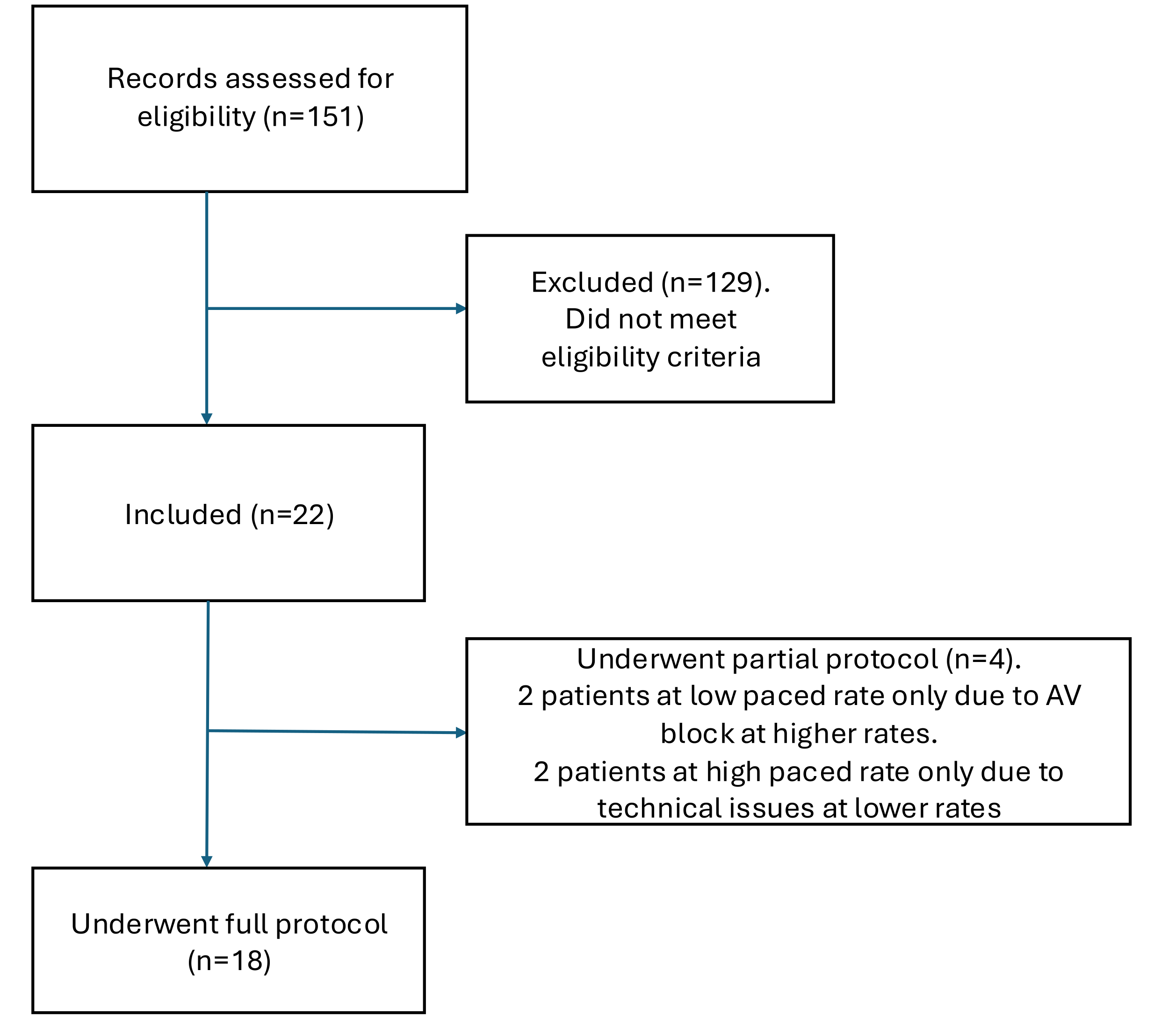

Supplement: Supplementary file 5 — Supplementary Fig. 4: Consort Diagram. [file PACE-48-1138-s004.tif]
